# Localized surface plasmon resonance-based abscisic acid biosensor using aptamer-functionalized gold nanoparticles

**DOI:** 10.1371/journal.pone.0185530

**Published:** 2017-09-27

**Authors:** Shun Wang, Wei Li, Keke Chang, Juan Liu, Qingqian Guo, Haifeng Sun, Min Jiang, Hao Zhang, Jing Chen, Jiandong Hu

**Affiliations:** 1 College of Mechanical and Electrical Engineering, Henan Agricultural University, Zhengzhou, China; 2 State Key Laboratory of Wheat and Maize Crop Science, Zhengzhou, China; 3 College of Science, Henan Agricultural University, Zhengzhou, China; 4 College of Agronomy, Henan Agricultural University, Zhengzhou, China; 5 College of Life Sciences, Henan Agricultural University, Zhengzhou, China; Institute of Materials Science, GERMANY

## Abstract

Abscisic acid (ABA) plays an important role in abiotic stress response and physiological signal transduction resisting to the adverse environment. Therefore, it is very essential for the quantitative detection of abscisic acid (ABA) due to its indispensable role in plant physiological activities. Herein, a new detection method based on localized surface plasmon resonance (LSPR) using aptamer-functionalized gold nanoparticles (AuNPs) is developed without using expensive instrument and antibody. In the presence of ABA, ABA specifically bind with their aptamers to form the ABA-aptamer complexes with G-quadruplex-like structure and lose the ability to stabilize AuNPs against NaCl-induced aggregation. Meanwhile, the changes of the LSPR spectra of AuNP solution occur and therefore the detection of ABA achieved. Under optimized conditions, this method showed a good linear range covering from 5×10^−7^ M to 5×10^−5^ M with a detection limit of 0.33 μM. In practice, the usage of this novel method has been demonstrated by its application to detect ABA from fresh leaves of rice with the relative error of 6.59%-7.93% compared with ELISA bioassay. The experimental results confirmed that this LSPR-based biosensor is simple, selective and sensitive for the detection of ABA. The proposed LSPR method could offer a new analytical platform for the detection of other plant hormones by changing the corresponding aptamer.

## Introduction

Plant endogenous hormones are a class of small molecular organic compounds produced by plants themselves. These endogenous hormones regulate all processes of plant growth and development, which are widely used in agriculture [1]. Among these hormones, abscisic acid (ABA) plays an important role in abiotic stress response and physiological signal transduction resisting to the adverse environment, such as drought, low temperature and high-salt stress environment [2]. So the quantitative determination of ABA is very essential for the physiological process of plants. Up to now, a number of analytical techniques have been developed for the detection of ABA, such as capillary electrophoresis [3], electrochemical immunosensors [4–6], high performance liquid chromatography [7] and liquid chromatography/mass spectrometry methods [8–11]. However, these methods have their inherent disadvantages, such as sophisticated instrument dependence and time-consuming procedures. Therefore, it is desirable to develop an accurate, sensitive and low-cost approach for ABA detection.

In the past decades, a great deal of attention has been paid to gold and silver nanomaterials in various research fields, such as biosensing [12–14], imaging [15], catalysis [16–18] and diagnostic [19]. These metal nanoparticles are very attractive due to their unique optical properties of localized surface plasmon resonance (LSPR). For example, a LSPR-based biosensor using gold nanostars has been developed for sensing of mercaptoundecanoic acid [20]. Also, a reusable troponin-T (TnT) immunoassay using gold nanorods (GNR) has been demonstrated to be extremely sensitive to the dielectric constant of the surrounding medium [21]. Additionally, various biosensors based on the combination of gold nanoparticles (AuNPs) and aptamers have been widely developed due to the high extinction coefficient of AuNPs and high specificity of aptamers [22–31]. In principle, the well-dispersed AuNP solution is red, whereas the aggregated AuNP solution appears purple or even blue owing to the strong distance-dependent properties of AuNPs. This phenomenon is attributed to their remarkable LSPR character of AuNPs [32, 33]. Moreover, aptamers are single-stranded DNA/RNA oligonucleotides selected in vitro by systematic evolution of ligands using exponential enrichment (SELEX) technique, which are well known as alternatives to antibodies with many incomparable advantages, such as small molecular weight, no immunogenicity and easy modification [34,35]. They exhibited specific recognition ability to target molecules, accompanied by their own conformational changes from random coil structures to G-quadruplex-like structure [29,36,37]. For the method using the aptamers and AuNPs, the the single-stranded DNA (ssDNA) aptamer with random coil structures can be adsorbed on the surface of AuNPs, protecting AuNPs from salt-induced aggregation. Differently, the ssDNA aptamer with G-quadruplex-like structures interact weakly with AuNPs and thus lose the ability to stabilize AuNPs [38,39]. Therefore, the conformational change of aptamer provides a good chance to achieve the LSPR detection of target using aptamer-functionalized AuNPs.

Recently, a specific 76-base aptamer for ABA has been successfully obtained by some researchers. Especially, after binding with ABA, the conformation of the aptamer of aptamer has changed to a G-quadruplex-like structure [40]. Based on this conformational change, a low-cost and rapid LSPR method for ABA detection is firstly proposed, which exploited aptamer as the specific recognition element and AuNPs as the probes. The originally free ABA aptamer is adsorbed on the surface of the AuNPs, which can stabilize the AuNPs against salt-induced aggregation. However, in the presence of ABA and salt, the aptamer change its conformation and specifically interact with ABA molecules, thereby resulting in the LSPR spectral changes of AuNPs and achieving the ABA detection.

## Experimental

### Materials and apparatus

Abscisic acid (ABA), Vitamin C (VC), beta-Carotene (β-Car), chloroauric acid and tris-sodium citrate were purchased from Sigma-Aldrich (USA). Methanol and sodium chloride were obtained from Sinopharm Chemical Reagent Co., Ltd. (Shanghai, China). The whole ABA aptamer, 5′- GCG GAT GAA GAC TGG TGT GAG GGG ATG GGT TAG GTG GAG GTGGTT ATT CCG GGA ATT CGC CCT AAA TAC GAG CAA C-3′, split aptamer fragment-1, 5′- GCG GAT GAA GAC TGG TGT GAG GGG ATG GGT TAG GTG GAG GTG-3′ (Sapt-1), and split aptamer fragment-2, 5′-GTT ATT CCG GGA ATT CGC CCT AAA TAC GAG CAA C-3′ (Sapt-2) were synthesized by Sangon Biotechnology Co., Ltd. (Shanghai, China). Fresh leaves of rice were provided by Agronomy College of Henan Agricultural University. Before use, the whole ABA aptamer and the split aptamer were dissolved in 100 mM PBS buffer solution (pH 7.0) and annealed at 95°C for five minutes. Ultra-pure water was used throughout the experiment.

The ultraviolet-visible (UV-Vis) absorption spectra were recorded by using an UV-Vis spectrophotometer with a spectral resolution of 1 nm (Nanjing Philes Instruments Co., Ltd. Nanjing, China).

### The synthesis of AuNPs

AuNPs were synthesized by the citrate reduction of HAuCl_4_ in aqueous solution according to the literature [41]. Briefly, 10 mL sodium citrate solution (38.8 mM) was quickly added into a boiling solution of 100 mL HAuCl_4_ (1 mM) with vigorous stirring. After the color of the solution changed to deep red, the solution was heated for an additional 20 min. Subsequently, the solution was cooled to room temperature and then stored in a clean tube at 4°C for further use. The morphology and particle size of prepared AuNPs were characterized by a JEM-2100 high resolution transmission electron microscopy (TEM). The synthesized Au NPs shows the absorption maximum at 520 nm with an extinction coefficient (2.52×10^8^ M^-1^ cm^-1^), and the concentration of Au NPs solution was about 7.51 nM [42].

### Pretreatment of ABA samples

The preparation procedures of ABA samples are listed as follows. First, 1.0 g fresh leaves of rice were cut into a number of small pieces and ground with liquid nitrogen, followed with a 20 min ultrasound treatment after adding 2 mL methanol-water-formic acid solution (15:4:1, v/v/v) and kept at -20°C for 24 h in the refrigerator away from light. Then, centrifugation at the speed of 10000 rpm was performed for 10 min at 4°C and the supernatant was kept in a 4 mL centrifuge tube for avoiding exposure to bright light. Extract for two times with 1 mL of methanol-water-formic acid (15:4:1, v/v/v) and then the supernatant was also kept in the 4 mL centrifuge tube. After that, all of the supernatants are dried in the vacuum evaporation devices at 30°C and then dissolved with 1.0 mL methanol-water-acetic acid (90:10:0.05, v/v/v). Finally, the supernatants were centrifuged at the speed of 10000 rpm at 4°C for another 10 min for future analysis.

### Detection procedure of ABA using LSPR sensor

Typically, 30 μL of 1 μM ABA aptamer was firstly mixed with 100 μL of AuNPs in a 0.6 mL plastic vial for 30 mins at room temperature. For the split aptamer, the concentration and corresponding volume were 2 μM and 15 μL, respectively. Subsequently, 100 μL of ABA samples in different concentrations were added into plastic vials. In order to obtain a final constant volume of 500 μL, 170 μL ultra-pure water were added. Then incubation were performed at 37°C for 45 min with a thermostat steam bath vibrator. After that, 100 μL of NaCl solution (500 mM) was added quickly into these vials. After two minutes, 300 μL of the resulting solutions were transferred into quartz micro cuvettes with 10-mm path-length for spectral analysis by using UV-Vis spectrophotometer.

## Results and discussion

### The LSPR detection principle for the detection of ABA

**Fig 1:**
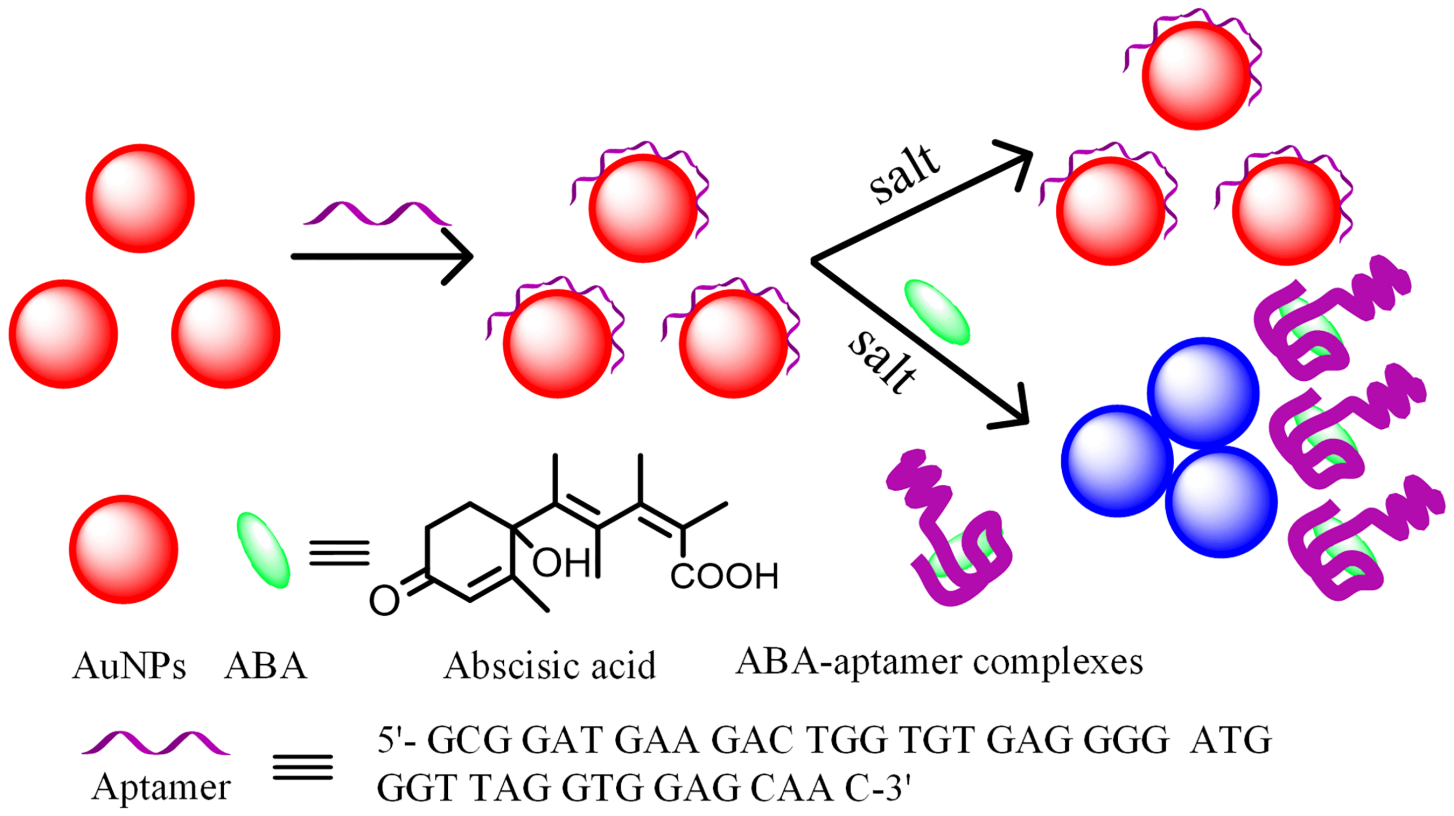
Schematic diagram of the LSPR method using aptamer-functionalized for the detection of ABA.

**Fig 2:**
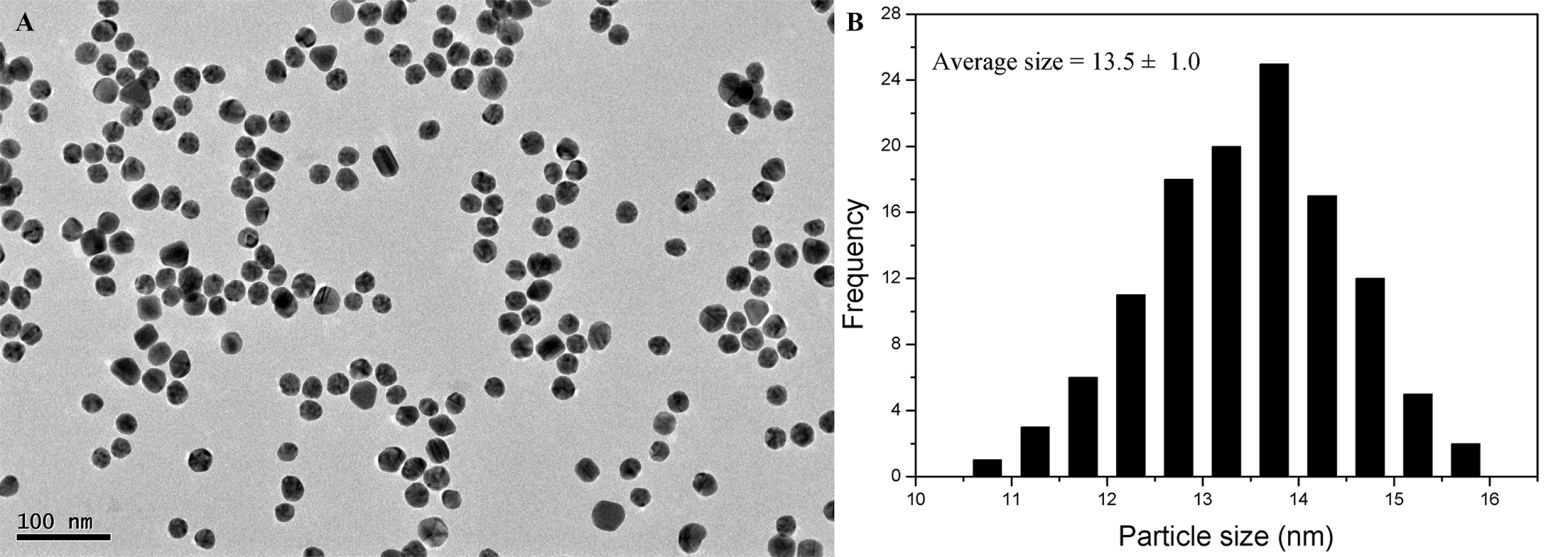
(A) TEM image of citrate-stabilized AuNPs and (B) the diagram of corresponding particle-size distribution.

The principle of the LSPR detection of ABA by using the aptamer-functionalized AuNPs is illustrated in Fig 1. The originally synthesized AuNPs with an average diameter of 13.5±1.0 nm were stable and well-distributed due to the repulsion interactions between citrate anions coated onto the surface of AuNPs (see Fig 2). Under this condition, the AuNP solution appears red. However, once the high-concentration NaCl solution was added, it screened the electrostatic repulsion between citrate anions. In this case, the AuNPs aggregated and exhibited a blue color, resulting in a red shift of LSPR peak of AuNPs. However, the AuNP solution were still kept in a well-dispersed state and appeared red due to the presence of free ABA aptamer which worked as an inhibitor against the NaCl-induced aggregation. The reason is that ssDNA with a random coil structure can be absorbed on the surface of AuNPs through the coordination interaction between the nitrogen atoms of the exposed bases and AuNPs, where the negative charges were genetated on the AuNPs surface, increasing their repulsion among AuNPs [43,44]. Once the target ABA was added into the aptamer-AuNP solution, the ssDNA conformation changed into a G-quadruplex-like structure and specifically bind with ABA, leading to the aptamer desorption from the AuNP surface and losing the ability to stabilize AuNPs. The interaction between aptamer and ABA decrease the amount of aptamer strands adsorbed on the surface of AuNPs through the conformational change and desorption of aptamer strands. Upon addition of a high-concentration of NaCl, negative charge on the surface of AuNPs will drastically reduce. In this case, the AuNPs aggregated immediately, resulting in the red shift of LSPR spectra and the color changes.

**Fig 3:**
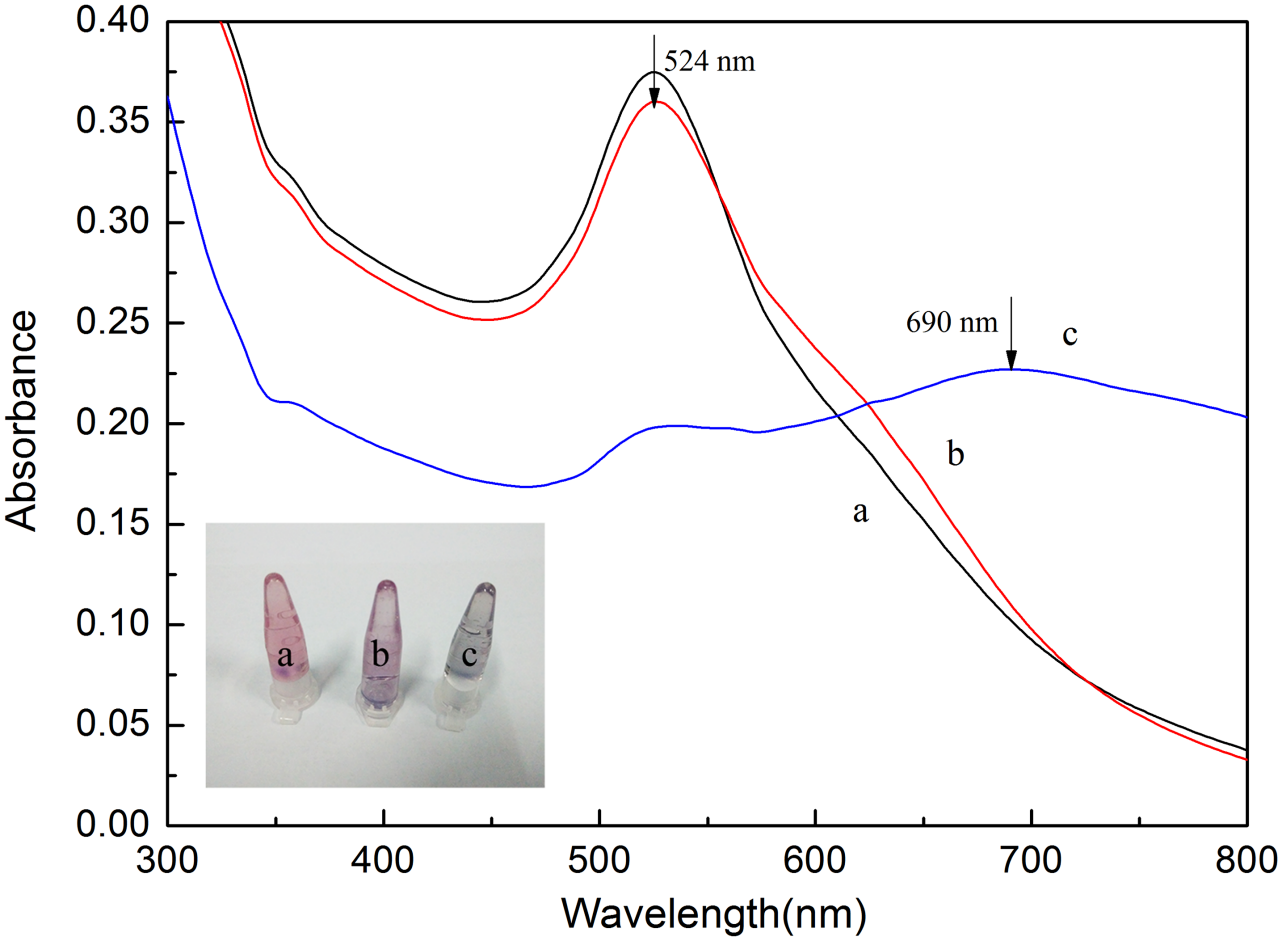
The LSPR absorption spectra of AuNP solutions obtained from the different reagents-added samples: a, aptamer+NaCl; b, aptamer+ABA (5 μM) + NaCl; c, NaCl. Inset: The corresponding photos of AuNP solutions with different samples.

To further demonstrate the possibility of the LSPR method for ABA detection, a series of UV-Vis spectra of AuNPs were taken under different experimental conditions (see Fig 3). When 100 mM NaCl was added into the AuNP solution, the color of the solution changed from red to blue (see inset in Fig 3, tube c) and the absorption peak intensity at 520 nm decreased and a new absorption peak at 700 nm appeared (shown in Fig 3, curve c). As expected, the aggregation effect was induced because the electrostatic repulsion between the ion-coated AuNPs was screened, resulting in the red shift of LSPR band. Differently, in the presence of ABA aptamer, the AuNP solution appear red (see the inset in Fig 3, tube a) and there existed only one peak at about 520 nm (shown in Fig 3, curve a), indicating that the AuNPs were well-dispersed. This can be ascribed to the fact that the ssDNA can protect AuNPs against the NaCl-induced aggregation. When the target ABA was added into the aptamer-AuNP solution, the conformation of aptamer changed into a G-quadruplex-like structure and then interacted with ABA, losing their ability to protect AuNPs from salt-induced aggregation under such a high ionic strength condition. Thus the color of the aptamer-functionalized AuNP solution changed into purple (see the inset in Fig 3, tube b). Meanwhile, the LSPR adsorption band of AuNP solution also showed red shift with a new absorption peak at about 620 nm (shown in Fig 3, curve b). These experimental results further prove that this aptamer-functionalized AuNPs can act as LSPR detection probes for ABA detection.

### The comparison experiments performed by the whole and the split ABA aptamer

**Fig 4:**
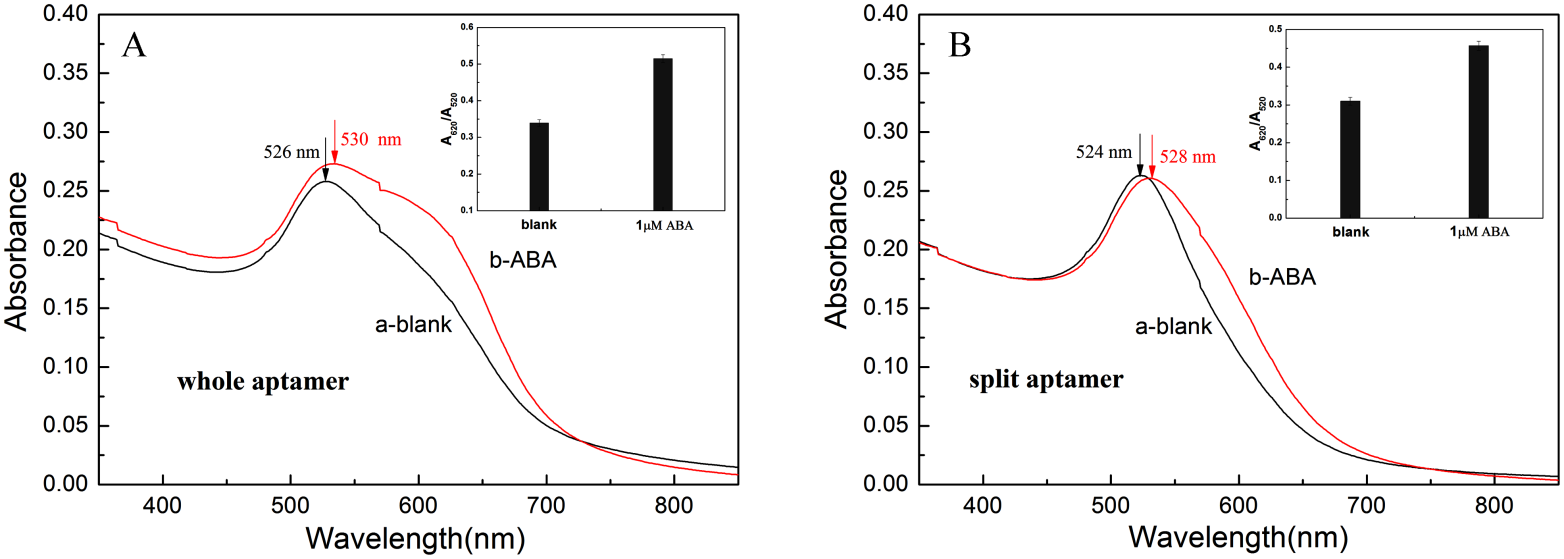
The LSPR absorption spectra of detecting target ABA using the whole aptamer (A) and the split aptamer (B) in the absence (a) and presence of ABA (b). Inset: The absorbance ratio at 620 nm and 520 nm (A_620_/A_520_) in the absence and presence of ABA.

To investigate the effect of the whole and split aptamer on the sensitivity of this LSPR detection of ABA, the whole 76-base ssDNA specific for ABA was divided into two short strands at the site of 42G-34G (see S1 Fig), namely Sapt-1 of 42-base ssDNA and Sapt-2 of 34-base ssDNA. From S1 Fig, the split site was selected just in the loop near the center of the sequence to cause the minimum effect on the complete conformation. The response value of Δ(A_620_/A_520_) (the difference between the absorption ratios A_620_/A_520_ calculated from experiment results performed in the presence and absence of ABA) of the whole and split aptamer were calculated to be 0.177 and 0.147, respectively (shown in Fig 4). The results demonstrated that the sensitivity obtained from the whole 76-base aptamer is higher than that from split aptamer. This may be explained from the fact that the binding probability of the split aptamer to the target molecules decreased.

### Optimization of experimental conditions

The aggregation and the LSPR spectra of AuNPs are highly related to the salt concentration, the concentration of aptamer, and the incubation time after adding the target of ABA into the AuNP-aptamer complexes. The whole aptamer was selected for all the following experiments and the Δ(A_620_/A_520_) was used to optimize the experimental conditions. First, different concentrations of 40 mM, 60 mM, 80 mM, 100 mM and 120 mM NaCl were studied, as shown in S1 Table. The results demonstrated that the Δ(A_620_/A_520_) reached the maximum value at the concentration of 100 mM. Thus, 100 mM of NaCl was chosen for the whole experiment. Subsequently, the effect of aptamer concentration was also investigated over the range of 20–80 nM and the experimental results were depicted in S2 Table. From S2 Table, it is noted that the maximum value of Δ(A_620_/A_520_) was obtained at the concentration of 60 nM. Thus, the concentration of aptamer was selected to be 60 nM. Finally, the incubation time was studied over the range of 20–60 mins, and the steady value of Δ(A_620_/A_520_) was reached at the time of 45 min (see S3 Table), which showed that the interaction could be completed within 45 min. Therefore, 45 min was selected as the optimized incubation time.

### LSPR detection of ABA using aptamer-functionalized AuNPs

**Fig 5:**
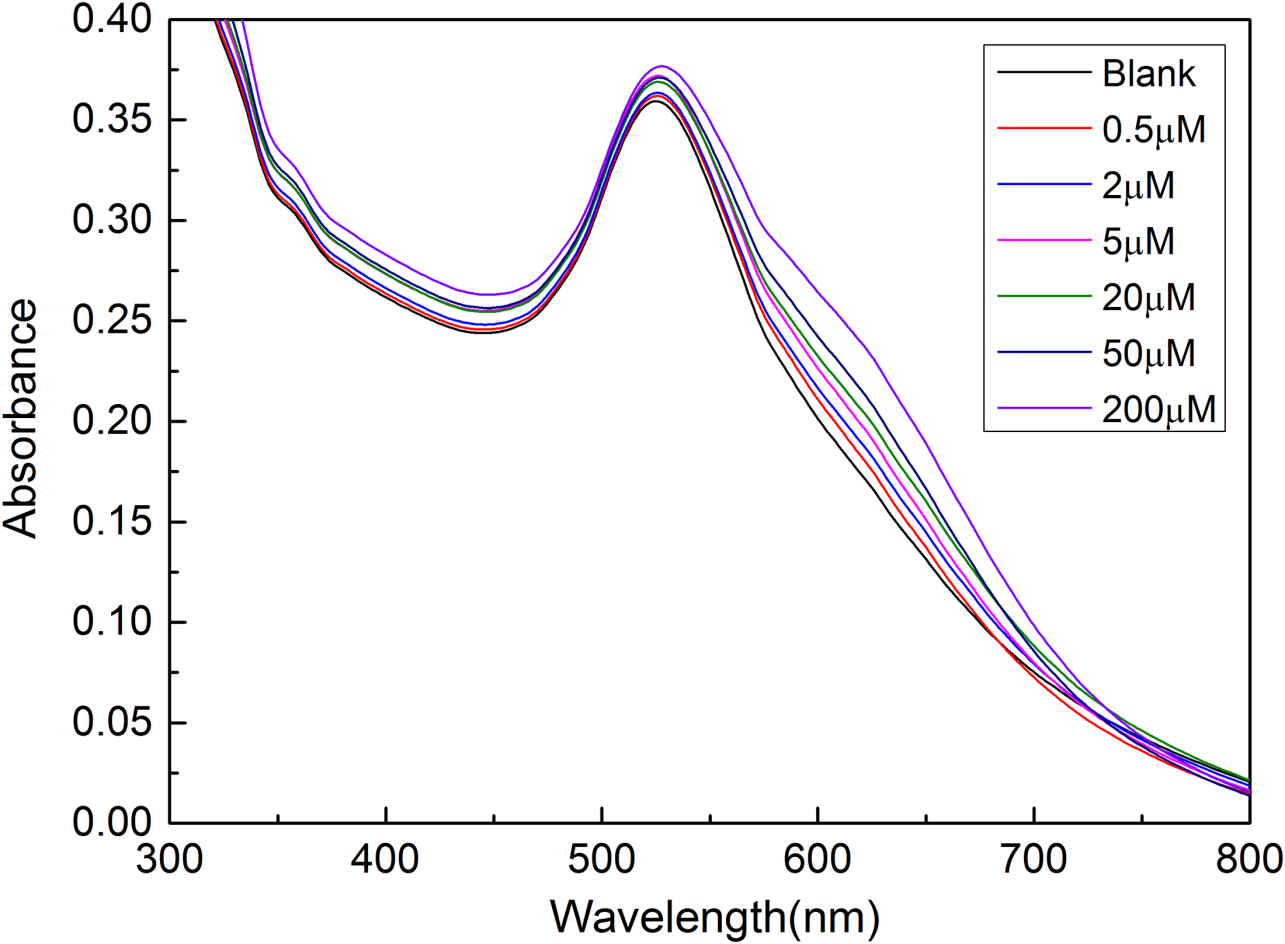
UV-Vis absorption spectra of aptamer-AuNP solution obtained from different concentrations of ABA samples. Experimental conditions: C_NaCl_ = 100 mM, C_aptamer_ = 60 nM.

**Fig 6:**
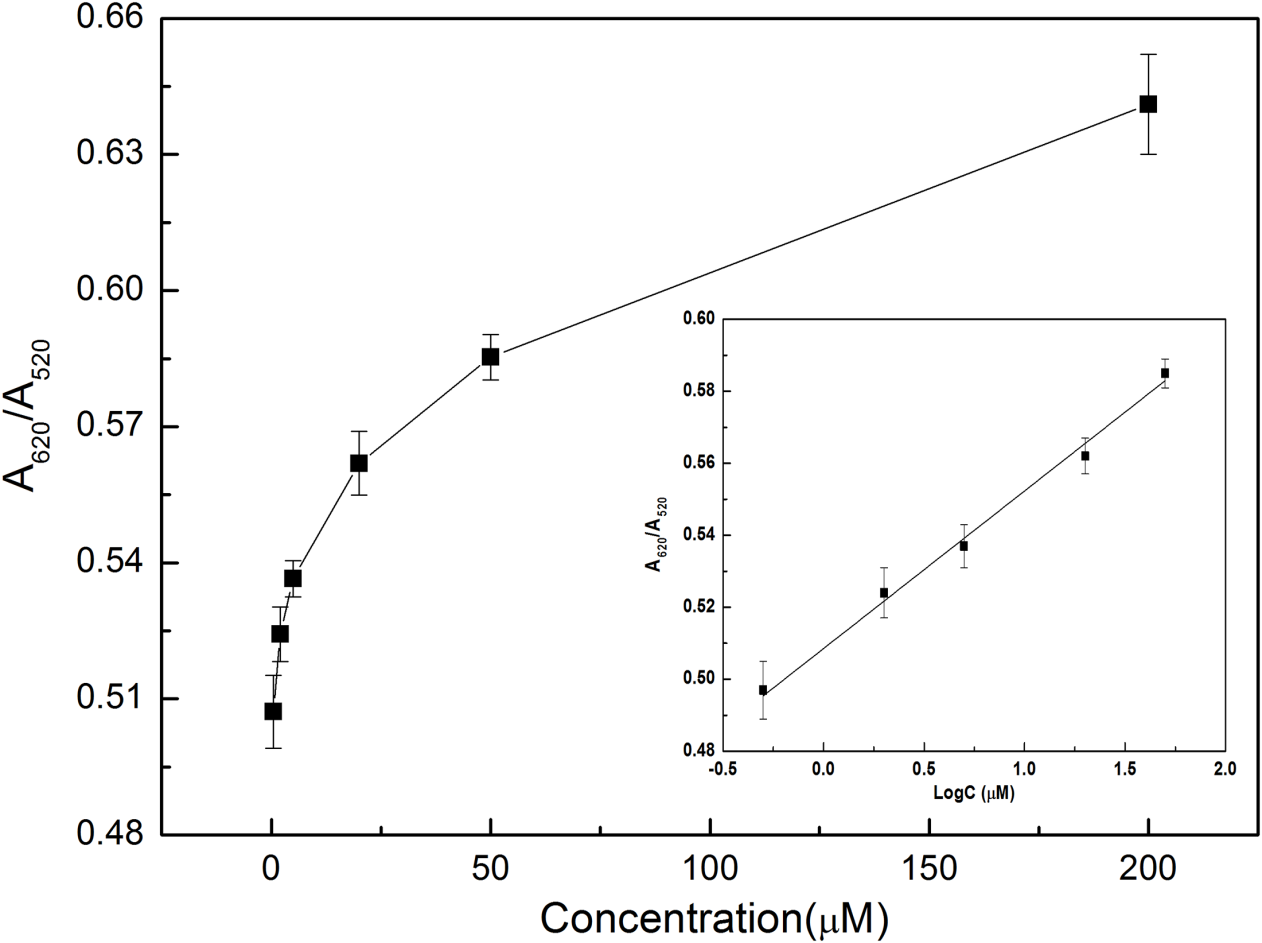
Calibration curve established from the absorbance ratio (A_620_/A_520_) using different concentrations of ABA samples. Experimental conditions were the same as in Fig 4.

To quantitatively detect ABA based on the proposed LSPR method using aptamer-functionalized AuNPs, a series of ABA solutions with different concentrations of 0.5 μM, 2 μM, 5 μM, 20 μM, 50 μM and 200 μM were sequentially added into the aptamer-AuNP solution for 45 min incubation, respectively. Under the above optimized experimental conditions, the absorption spectra were recorded by a UV-Vis spectrophotometer (see Fig 5). As can be seen from Fig 5, with the increase of the concentration of ABA, the absorption peak intensity at 520 nm have no obvious change, while the absorption peak intensity at 620 nm increased gradually. Based on the recorded absorption spectra, a relationship curve between the absorption ratio (A_620_/A_520_) and the concentration of ABA was established. As shown in Fig 6, the absorption ratio of A_620_/A_520_ increased with the increase of the concentration of ABA in the range of 0.5–200 μM. As depicted Fig 6 (Inset), a calibration curve was established by using the fitting equation *Y* = *A*_620_/*A*_520_ = 0.5119+0.0390×log *C* with a correlation coefficient R of 0.992 excluding the concentration of 200 μM, where *Y* is the ratio of A_620_/A_520_ and *C* is the concentration of ABA (μM). The limit of detection of 0.33 μM was calculated from the empirical formula expressed as 3*σ*/*k*, where *σ* is the standard deviation and *k* is the slope of the fitting equation. Compared with reported techniques (see S4 Table), these experimental results show that the LSPR-based biosensor using aptamer-functionalized gold nanoparticles was highly effective for ABA detection at a relatively low concentration without the use of expensive instrument and antibody.

### Selectivity of this LSPR detection method

**Fig 7:**
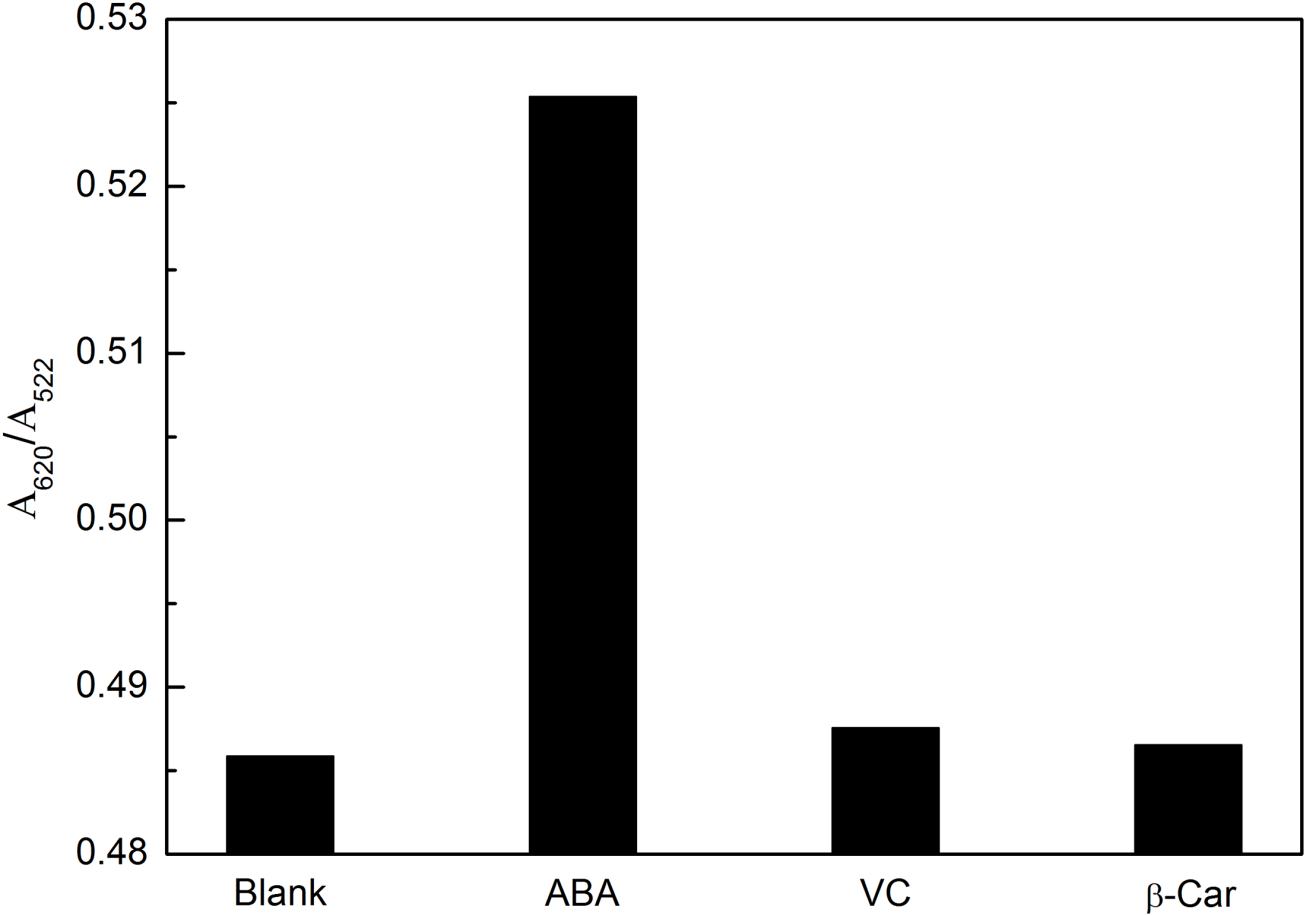
Selectivity of this LSPR method for detection of ABA. Experimental conditions were the same as in Fig 4. ABA: 2 μM; Other compounds: 10 μM.

Some other ingredients in the extracts of real samples might interfere with the detection of ABA. The selectivity of this method was evaluated by detecting some compounds that existed in the extracts, such as VC and β-Car. As can be observed in Fig 7, comparing with the A_620_/A_520_ of blank sample, 2 μM of ABA exhibits a noticeable increase, while only a minor change occurs even for the concentration of 10 μM of the VC or β-Car. These results indicate that this approach exhibit good specificity for the detection of ABA.

### Detection of ABA in real samples

To further validate this proposed LSPR method in practical applications, fresh leaves of rice were chosen for this experiment. For comparison, the ABA content of these samples was also measured by using enzyme-linked immunosorbent assay (ELISA) method. The results were summarized in Table 1. Comparing with the ELISA method, the calculated maximum relative error is 7.93%, which exhibits an acceptable agreement between the two methods. From the experimental results, it can be concluded that this proposed method could provide a potential method for the detection of other plant hormones, such as gibberellins, indoleacetic acids and so on.

**Table 1:** Analytical results of ABA in fresh leaves of rice.

Sample	Found by present method (ng·g^-1^)	Found by ELISA (ng·g^-1^)	Relative error compared with ELISA (%)
**1**	105.1954	97.4663	7.93
**2**	105.3229	97.6893	7.24
**3**	112.7154	107.5529	6.59

## Conclusions

In this work, we have successfully developed a novel and specific method based on LSPR using the aptamer-functionalized AuNPs for quantitative detection of ABA without the requirement of complicate and expensive instruments and antibody. In the presence of ABA, the aptamers interact with ABA and lose the ability to stabilize AuNPs against NaCl-induced aggregation, resulting in the red shift and the LSPR spectral changes of AuNPs. In addition, the obtained experimental results show a good linear relationship between the absorption ratio of A_620_/A_520_ and the concentration range of log*C* from 5 × 10^−7^ M to 5 × 10^−5^ M with a detection limit of 0.33 μM. Some interferents such as VC and β-Car showed just no obvious interference in the detection of ABA. The practical use of this assay has been demonstrated by its application to detect ABA from fresh leaves of rice with the relative error of 6.59%-7.93% compared with ELISA bioassay. The versatility of this LSPR assay makes this technology a powerful method for the detection of ABA. The proposed method is also expected to be readily applied to the detection of other plant hormones by only changing the appreciate aptamer.

## Supporting information

S1 Fig Secondary structure of the ABA aptamer selected in literature using a web-based tool *mfold*. The red arrow indicates the split site.
(TIF)

S1 Table The relationship between the concentration of NaCl and Δ(A_620_/A_520_) under the condition of the aptamer concentration of 60 nM and the incubation time of 1 h. (DOC)

S2 Table The relationship between the concentration of aptamer and Δ(A_620_/A_520_) under the condition of the NaCl concentration of 100 mM and the incubation time of 1h. (DOC)

S3 Table The relationship between the incubation time and Δ(A_620_/A_520_) under the condition of the NaCl concentration of 100 mM and the aptamer concentration of 60 nM. (DOC)

S4 Table Comparison of the analytical techniques for ABA detection between the reported techniques and this work. (DOC)
