# Supplementary figures and images for: Localized surface plasmon resonance-based abscisic acid biosensor using aptamer-functionalized gold nanoparticles
Source: PLoS One. 2017 Sep 27;12(9):e0185530. doi: 10.1371/journal.pone.0185530 (PMC5617216; doi:10.1371/journal.pone.0185530)

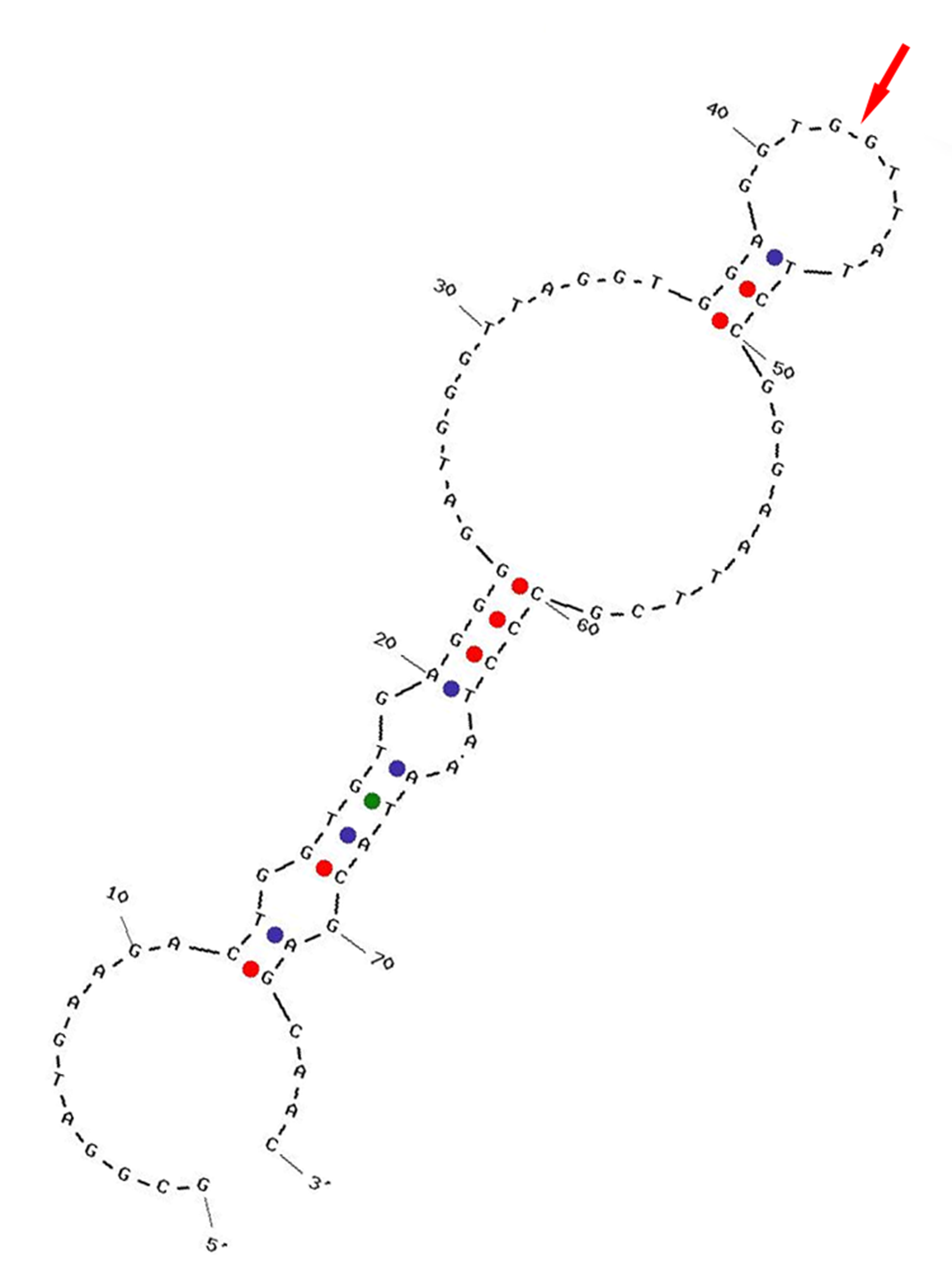

Supplement: S1 Fig — The red arrow indicates the split site. (TIF) [file pone.0185530.s001.tif]
