# Supplementary material for: Localized surface plasmon resonance-based abscisic acid biosensor using aptamer-functionalized gold nanoparticles
Source: PLoS One. 2017 Sep 27;12(9):e0185530. doi: 10.1371/journal.pone.0185530 (PMC5617216; doi:10.1371/journal.pone.0185530)
Supplement: S1 Table — (DOC) [file pone.0185530.s002.doc]

**S1 Table. The relationship between the concentration of NaCl and Δ(A_620_/A_520_) under the condition of the aptamer concentration of 60 nM and the incubation time of 1 h**.

| NaCl/mM | 40 | 60 | 80 | 100 | 120 |
| --- | --- | --- | --- | --- | --- |
| Δ(A_620_/A_520_) | 0.0010 | 0.0111 | 0.0233 | 0.0547 | 0.0489 |
