# Supplementary material for: Localized surface plasmon resonance-based abscisic acid biosensor using aptamer-functionalized gold nanoparticles
Source: PLoS One. 2017 Sep 27;12(9):e0185530. doi: 10.1371/journal.pone.0185530 (PMC5617216; doi:10.1371/journal.pone.0185530)
Supplement: S2 Table — (DOC) [file pone.0185530.s003.doc]

**S2 Table. The relationship between the concentration of aptamer and Δ(A_620_/A_520_) under the condition of the NaCl concentration of 100 mM and the incubation time of 1 h**.

| Aptamer/nM | 20 | 40 | 60 | 70 | 80 |
| --- | --- | --- | --- | --- | --- |
| Δ(A_620_/A_520_) | - | 0.0529 | 0.0546 | 0.0553 | 0.0557 |

Note: “-”, for 20 nM aptamers without ABA molecules, the AuNPs aggregated immediately and the color appeared blue when NaCl solution was added into the AuNPs solution.
