# Supplementary material for: Localized surface plasmon resonance-based abscisic acid biosensor using aptamer-functionalized gold nanoparticles
Source: PLoS One. 2017 Sep 27;12(9):e0185530. doi: 10.1371/journal.pone.0185530 (PMC5617216; doi:10.1371/journal.pone.0185530)
Supplement: S3 Table — (DOC) [file pone.0185530.s004.doc]

**S3 Table. The relationship between the incubation time and Δ(A_620_/A_520_) under the condition of the NaCl concentration of 100 mM and the aptamer concentration of 60 nM.**

| Incubation time/min | 20 | 30 | 45 | 60 |
| --- | --- | --- | --- | --- |
| Δ(A_620_/A_520_) | 0.0231 | 0.0476 | 0.0548 | 0.0549 |
