# Supplementary material for: Localized surface plasmon resonance-based abscisic acid biosensor using aptamer-functionalized gold nanoparticles
Source: PLoS One. 2017 Sep 27;12(9):e0185530. doi: 10.1371/journal.pone.0185530 (PMC5617216; doi:10.1371/journal.pone.0185530)
Supplement: S4 Table — (DOC) [file pone.0185530.s005.doc]

**S4 Table. Comparison of the analytical techniques for ABA detection between the** reported techniques and this work

| **Techniques** | **Advantages** | **Limitations** | **Refs** |
| --- | --- | --- | --- |
| Capillary electrophoresis | - Low sample consuming. - High separation efficiency with LOD of 55 nM. | - Small variations in pH have a greater impact in CE | [3] |
| Electrochemical immunosensors | - Highly affinity due to the used ABA antibody | - Time-consuming sample-handling steps | [5] |
| HPLC/HPLC-MS | - HPLC and HPLC-MS give the ability to distinguish between two plant hormones with LOD of 0.4-2 nM. | - Trained operators and expensive equipment | [7,8] |
| LSPR-based biosensor (this work) | - Visualized with naked eyes. - Without expensive instrument. - Aptamers with small molecular weight, no immunogenicity and easy modification. | - The affinity of aptamer to target can affect the detection sensitivity. | This work |
